# Cancer Targeting and Drug Delivery Using Carbon-Based Quantum Dots and Nanotubes

**DOI:** 10.3390/molecules23020378

**Published:** 2018-02-10

**Authors:** Joel Pardo, Zhili Peng, Roger M. Leblanc

**Affiliations:** 1 Department of Chemistry, University of Miami, 1301 Memorial Drive, Coral Gables, FL 33146, USA; jxp1142@miami.edu; 2 College of Pharmacy and Chemistry, Dali University, Dali 671000, Yunnan, China

**Keywords:** carbon dots, carbon nanotubes, drug delivery, doxorubicin, gemcitabine, transferrin, folic acid, hyaluronan

## Abstract

Currently cancer treatment is in large part non-specific with respect to treatment. Medication is often harsh on patients, whereby they suffer several undesired side effects as a result. Carbon-based nanoparticles have attracted attention in recent years due to their ability to act as a platform for the attachment of several drugs and/or ligands. Relatively simple models are often used in cancer research, wherein carbon nanoparticles are conjugated to a ligand that is specific to an overexpressed receptor for imaging and drug delivery in cancer treatment. These carbon nanoparticles confer unique properties to the imaging or delivery vehicle due to their nontoxic nature and their high fluorescence qualities. Chief among the ongoing research within carbon-based nanoparticles emerge carbon dots (C-dots) and carbon nanotubes (CNTs). In this review, the aforementioned carbon nanoparticles will be discussed in their use within doxorubicin and gemcitabine based drug delivery vehicles, as well as the ligand-mediated receptor specific targeted therapy. Further directions of research in current field are also discussed.

## 1. Introduction

Carbon dots (C-dots) are emerging nanomaterials with incredible versatility. First discovered in 2004 by Scrivens and co-workers [1], C-dots have had far reaching implications for chemistry, engineering, biology, medicine, and several other fields [2]. Applications for this emerging material can be found in areas such as electronics [3], sensor development [4], polymers [5], and imaging [6,7], of particular interest is the application of C-dots in the medical fields [8]. C-dots are becoming a prevalent platform for attachment of receptors alongside chemotherapy drugs due to the presence of rich surface functional groups (i.e., carboxylic and amino groups) [9,10]. C-dots have attracted such attention in part due to the simplistic materials and plethora of different methods of synthesis; whereby, complicated machinery is often not necessary [11,12]. Synthesis methods range from using strong acids to functionalize carbon powder (“top-down” approach) to heating above the melting temperature using technology as simple as a conventional microwave (“bottom-up” approach) [13,14]. The composition of C-dots may vary slightly due to different methods in synthesis [15,16]. These variations in synthesis could lead to differences in the fluorescence, size, and activity. Photoluminescence is perhaps the most well studied aspect of C-dots, lending itself to straightforward characterization once conjugation with target molecule is achieved. C-dots are usually excitation-wavelength dependent; their emission spectra typically range from the mid 300 nm to as high as 700 nm [17,18].

In conjunction with the development of material science, it is of no surprise that research towards drug development has grown and will continue to grow at a tremendous rate. An over 400% increase in cancer survival by 2022 is projected when compared to 1977, the majority of growth occurring in the long-term (15+ years) survival of patients [19]. This drastic increase is largely due to growing research; nonetheless, the adaptability and sophisticated heterogeneity cancer possess allow it to develop drug resistance during treatment, reducing therapeutic effects [20]. Most often several drugs are prescribed to allow for the possibility of tumor resection. Most anticancer drugs cause tremendous damage not only to the cancer cells for which its administration was intended, but also to unrelated healthy somatic cells. Such damage causes many of the common symptoms associated with chemotherapy patients (hair loss, pale skin, vomiting, etc.) [21]. Improving targeting efficiency of cancer cell lines with anticancer drugs is one of the dominant frontiers of cancer research. By specifically targeting cancer cells with the aid of novel nanomaterials, it is expected that overall drug dosages can be lowered due to higher drug efficacy, causing decreased side effects and increased patient quality of life [22].

To this end C-dots are nontoxic and can inhibit human insulin fibrillation [13]. They have also been reported to efficiently suppress cancer cell in vitro and can inhibit Hep G2 growth, a liver cancer [23,24]. Similar inhibition has been seen in MCF-7 and MDA-MB-231 cancer cells (breast cancer) where the cause is believed to be due to generation of great amounts of reactive oxygen species (ROS) [24]. C-dots are serving as a similar platform for conjugation as widely used polymers such as polyethylene glycol (PEG); however, C-dots may provide higher receptor binding affinity and improved cell penetration when conjugated to an antibody [25]. Moreover, conjugated nanoparticles have shown the ability to be internalized through receptor-mediated endocytosis and accumulate in cells without being recognized by P-glycoprotein [26]. P-glycoprotein is one of the main contributors to drug resistance in cells. Furthermore, C-dots have shown to be able to bypass the blood-brain barrier in zebrafish models, a formidable obstacle for developing efficient treatments for brain-related diseases such as Alzheimer’s diseases [27]. Several papers have been published wherein an overexpressed receptor in a specific cancer cells is chosen to be covalently attached to C-dots alongside an anticancer drug, which constitutes a basic nano-delivery system. These kinds of systems can accumulate anti-tumor agents at the tumor sites due to enhanced permeability and retention effect [2,28,29].

Along the same vein carbon nanotubes (CNTs) have also emerged as potent nanocarrier with ever-growing popularity. CNTs were discovered by Dr. Iijima in 1991 and possess many similar qualities to C-dots as part of the carbon-based nanoparticle family [30]. These particles are nontoxic, have excellent optical properties, and can be quite small, with a strong capacity to be attached to other elements or particles for further functionalization [31]. Certain CNTs within drug delivery systems have the similar benefit to C-dots in having a well-studied release mechanism, wherein, acidic environment of tumor facilitates drug release [32]. The degree of functionalization within the surface of CNTs may be altered and thus the amount of drug released could be more accurately controlled [33,34]. As a result, their application in drug delivery systems has also been broadly studied.

Significant factors affecting the design of targeted drug delivery systems include an efficient means of delivery, conservation of drug bioactivity, and the enhancement of drug loading and release kinetics toward the drug targets [35]. In this paper we will first explore the commonly attached anti-cancer agents, using doxorubicin (Dox), and gemcitabine (gem) as examples; and then explore future possibilities of dual drug conjugates in carbon nanoparticle mediated delivery systems. Conjugation and characterization methods will be discussed for each case alongside the mechanism of action and overall effectiveness. Additionally, recurring ligands chosen for conjugation in carbon based drug delivery systems (transferrin, folic acid, and hyaluronan) will be subject to examination in a similar methodology as aforementioned in drug attachment. Finally, a brief overview will be given, followed by challenges faced, possible areas of interest, and overall outlook of the field.

## 2. Drug Usage and Resistances in Cancer Cells

Drugs chosen for cancer treatment must cause significant harm to the targeted cells, and to this end certain chemotherapy agents’ vehicle of action may be more effective on certain tumors over others. Removal of cancer stem cells is necessary in order to achieve a successful cancer free prognosis. Yet these stem cells have the ability of activating multiple drug resistance transporters which may lead to a constitutively drug-resistant cell [36]. More troubling is how some tumor cells show the capability of becoming a cancer stem cell in the absence of other cancer stem cells [37]. In this area nano delivery vehicles may prove more successful than other means of treatment such as conventional chemotherapy, as the higher percentage of drug reaching tumor cells in these systems allows for an increased instance of apoptosis to be achieved. The capability to overcome multiple drug resistance transporters have been shown in in vitro testing; however, in vivo applications have proven to be a true challenge due to effects elsewhere wherein premature inactivation of the drug may occur due to non-tumor cell drug resistances [36]. Thus, there is a need to overcome complications arisen from in vivo testing. Nonetheless, well-designed drug delivery systems are showing progress towards overcoming such pervasive issues. It should be noted, however, that many drug resistance mechanisms are not fully understood and subject ongoing research and debate [38].

To conjugate a drug to a carrier, certain criteria must be met. Of which the overall structure of the drug is foremost. A suitable functional group is often desired for successful attachment to C-dots. In covalent conjugation approaches, drugs are most often chosen containing free amines or carboxylic groups [39,40,41,42]. As a result, the relative ease of conjugation yields a high drug loading percentage. Covalent conjugation of drugs with carriers has advantages such as high loading yield, better controllability; however, it also faces challenges such as slow drug release and may lead to a new drug entirely ineffective due to conjugation. Finally, the drug candidates must be readily characterized once conjugated and purified to ensure accurate testing. To this end commonly chosen drugs for conjugation include Dox and gem; however, other anticancer agents such as paclitaxel, docetaxel, genes, and others are also seen in conjugation with a drug delivery system to improve their bioavailability [34,43]. A selective list of the drug delivery vehicles will be mentioned in this paper is shown below in Table 1.

### 2.1. Doxorubicin-Loaded Carbon-Based Nanoparticles

Doxorubicin (Dox) intercalates into the DNA and inhibits macromolecular synthesis [57,58,59]. Dox stabilizes topoisomerase II after DNA has been cut during replication, leading to overall cell degradation [57]. Dox is commonly prescribed to treat certain types of lymphomas, sarcomas, and leukemia [58], however, many researchers have found success using Dox in other cancers such as breast cancers and brain tumors [41,59]. It is commonly sold under the trade name Adriamycin. Dox has found great efficiency in targeting and eliminating cancer cells and is highly prized for its structure and ease of conjugation. Several C-dots related studies have been published targeting cancers of various types, many of which with promising result [39,40,41]. Yang and colleagues found that C-dots-Dox complexes could efficiently induce apoptosis in human lung adenocarcinoma cells [44]. The in vivo therapeutic efficacy of this conjugate was investigated in an A549 xenograft nude mice model wherein the complexes showed an enhanced ability to inhibit tumor growth compared to the free drug alone. In another study, it was found that C-dots and Dox make for an ideal drug release profile at physiological and slightly acidic pHs, following first order release kinetics. Furthermore, the receptor mediated delivery proved to lower the toxicity in normal cells [14]. Such a mechanism would likely find success in tumors of the early gastrointestinal tract or in patients with acidosis, wherein pH is lower. In a report by Wang and co-workers, it was found that C-dots-Dox conjugates within the HeLa cell separated after 6 h and entered the HeLa cell nuclear regions after 8 h in incubation [14,54].

**Figure 1:**
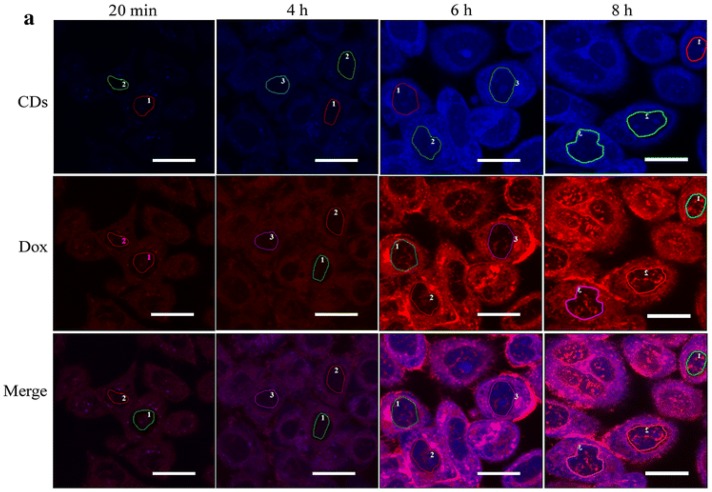
Confocal microscopy demonstrates Dox is released from C-dots-Dox conjugate at 6 h post treatment inside the cell, wherein, the conjugate regains the fluorescent capacity; the *scale bar* is 20 μm (reproduced with permission from [14]).

In the same study, Wang and co-workers found that the fluorescence of both C-dots and Dox was turned off in their conjugated form; however, following separation of the C-dots-Dox conjugate within the tumor cell, both the fluorescence of the C-dots and Dox were restored, which could be seen under confocal microscope (Figure 1), this indicates that pH dependent release is a reliable method to release attached conjugate system [14]. Dox has also been used in pediatric glioblastoma cell lines with noticeable effects on the tumors [41].

**Figure 2:**
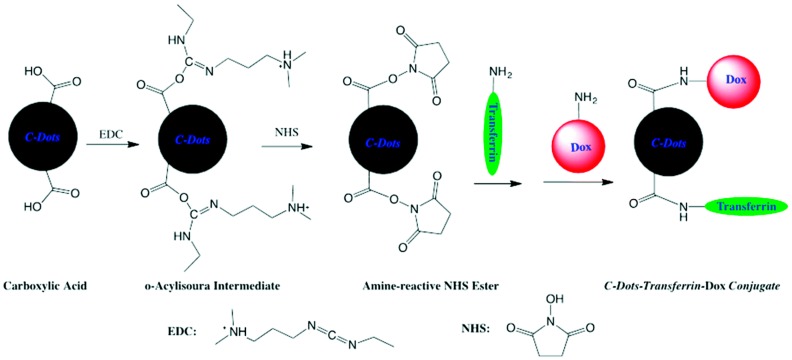
Conjugation of C-dots and Dox using EDC/NHS alongside transferrin attachment for drug delivery (reproduced with permission from [41]).

Methods to bind Dox to its carrier include covalent bonding between the functional groups of Dox and the carriers. Noncovalent conjugation through passive adsorption of Dox to the carriers has also been widely reported. The loading percentage could reach as high as 260% as reported by Sun et al. [45]. Dox is rich in sp^2^-hybridized carbons which allows for efficient loading onto C-dots using π-π stacking interaction, since C-dots can also be rich in sp^2^-hybridized carbons [60]. Dox is easily conjugated to C-dots through the formation of an amide bond between amine of dox and functionalized C-dots (Figure 2) [41]. In this reaction, C-dots were first activated by 1-ethyl-3-(3-dimethylaminopropyl)carbodiimide (EDC) and *N*-hydroxysuccinimide (NHS), after which dox was attached to C-dots by forming a stable chemical bond between the two. Such a method leads to a very stable product, which may mean a decreased amount of drug release or longer time period for release within the cancer cell. 

Fluorescence spectroscopy is most commonly used method of characterizing the Dox-C-dots conjugates, as both possess photoluminescence. C-dots most commonly have peaks around the high 300 nm to mid-400 nm depending on the synthesis method. By comparison, Dox has a peak at roughly 600 nm making characterization via this method fairly straightforward [14,41,44,46,61]. UV-Vis spectroscopy displays similar results as aforementioned with Dox having an absorption peak at 480 nm and most C-dots in the mid to late 300 nm. Furthermore, Dox possesses a positive zeta potential whereas most C-dots have a negative potential and upon conjugation a noticeable shift tends to occur [41,62,63].

**Figure 3:**
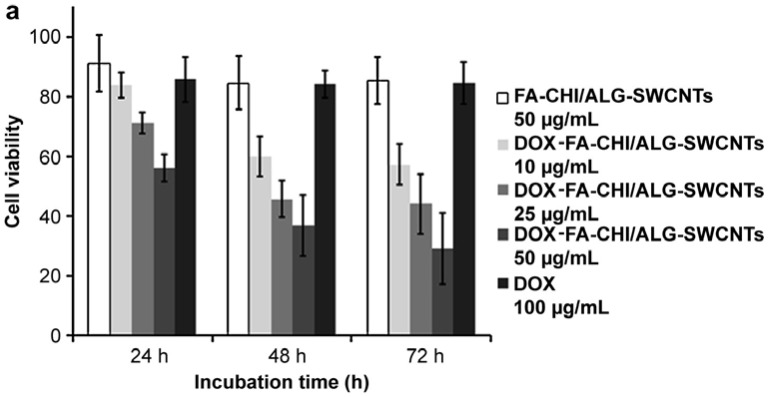

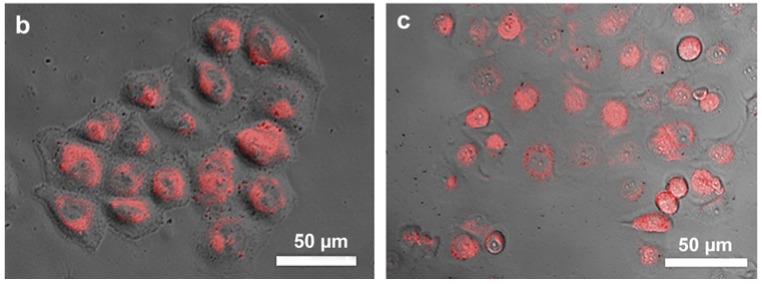
(**a**) Effectiveness of the CNTs-Dox conjugates within folic acid covered chitosan demonstrates the increased potency of nanocarrier systems as compared to free dox alone within HeLa cells. (**b**) Free Dox causes limited cell death as compared to (**c**) where cells noticeably begin to lose their structure and apoptosis (reproduced with permission from [34]).

Similarly, success in the use of CNTs for drug delivery has also been demonstrated in several reports [32,34,51]. Certain CNTs may provide excellent fluorescent capabilities or possess some pro-apoptotic capabilities; however, some CNTs may lack stability within a cell. The study by Huang et al. demonstrated a means to overcome such obstacles by encapsulating the CNTs-Dox conjugate within a folic acid-conjugated chitosan, thereby providing increased stability within the cationic chitosan [50]. In a different approach by Satyajit et al., a CNTs drug delivery system was formed by loading with hyaluronan and Dox [51]. The system demonstrated interesting results in their in vivo studies, wherein, some mice given a load of free Dox and another subset were given a load of CNTs functionalized with PEG to form a CNTs-hyaluronan-Dox conjugate system. Mice given the drug delivery system showed no outward changes and had remarkably reduced toxicity; by comparison, mice treated only with Dox demonstrated higher levels of toxicity, weight loss, and sluggishness. This demonstrated the potential of CNTs in improving drug efficacy and quality of life. In the same vein CNTs conjugated to Dox and inserted into folic acid attached chitosan at 50 μg/mL were able to decrease tumor cell viability the same degree compared to 100 μg/mL of Dox alone (Figure 3a) [34]. Improved apoptosis is visible and can be seen from Figure 3b,c within the free drug and drug delivery vehicle, respectively. The wide array of published papers indicate Dox as a flexible and potent anti-cancer agent with the ability to cause cellular apoptosis in nearly any cancer cell line and allow for a timely target delivery.

### 2.2. Gemcitabine-Loaded Carbon-Based Nanoparticles

**Figure 4:**
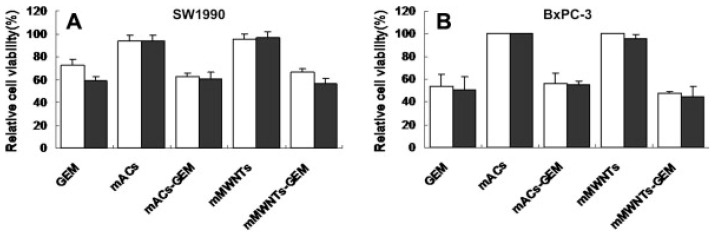
Cell viability in SW1990 cells (**A**) and BcPC-3 cells (**B**), when treated with magnetic multi-walled carbon nanotube (MWNTs)-Gem conjugates and magnetically activated carbon particle (mAC)-Gem conjugates show similar results as free Gem alone at concentrations of 1 ug/mL and 2.5 ug/mL respectively (reproduced with permission from [52]).

Gemcitabine (Gem) is a commonly prescribed medication for several different kinds of cancers. To this end it is highly versatile; in the medical practice it may be prescribed to patients suffering from mesotheliomas, pancreatic cancers, lung cancers, bladder cancers, cervical cancers, ovarian cancer, and several others [64]. Commonly marketed under the name Gemzar, it is a nucleoside analog of cytidine. It attaches to the growing DNA chain during replication and arrests its growth leading to cell apoptosis. Furthermore, gem also targets ribonucleotide reductase wherein deoxyribonucleotides are fabricated, thereby leading to cell death [65]. It has been found to be particularly effective against pancreatic cancers, especially against those underwent successful tumor resections [66]. Carbon nanoparticles have shown to prevent metastasis as well as impede the growth of tumors once successfully conjugated to gem and made into a drug delivery vehicle [31,42,52]. Regression of lymph node metastasis was even found in the study by Yang et al. [52]. Furthermore, in the same study magnetic multi-walled carbon nanotubes (MWCNTs) were found to be more effective at this than magnetically activated carbon particles, as shown in Figure 4 [52]. Interestingly, an external magnetic field can be applied to increase nanoparticle aggregation in certain areas and thereby increasing drug absorption in mice [61]. Gem is creating much excitement in terms of results as compared to the more commonly used Dox conjugate; however, as will be discussed, its characterization proves some difficulty as compared to Dox.

The free amine on Gem is often used as a handle for conjugation with drug carriers, however, it should be noted that this amine is attached to an aromatic ring for which, in principle, the expected overall yield would be lowered. Gem can be conjugated in a similar way as aforementioned in Dox conjugation methods, wherein EDC and NHS are used to activate the carboxylic groups on the C-dots for attachment to gem. π-π stacking conformation with the SWCNTs has been proposed in successfully conjugated nanotubes. In the study reported by Arsawang et al. they proposed the location of gem to be within a conjugated SWCNTs with simulated molecular dynamics [67]. Gem offers some difficulty when it comes to characterizing as commonly used methods such as fluorescence and UV-Vis suffer due to the lack of photoluminescence in Gem and overlap in absorption between C-dots and Gem. To this end, NMR is commonly implemented. Mass spectroscopy has also been speculated to be a successful characterization method. Infrared spectroscopy can be used as an efficient characterization method as well [68].

### 2.3. Other Drug Delivery Systems

Although Dox and Gem show promise as anti-cancer agents and attract most interest in the context of carbon nanoparticles based drug delivery systems development, other anti-tumor drugs have also found enhanced success when conjugated to C-dots and CNTs. C-dots conjugated to paclitaxel and hyaluronan unsurprisingly demonstrated the ability to kill tumor cells; C-dots used in the study were able to use near infrared light to image the tumor and follow its progression, as well as release paclitaxel in a pH dependent manner [69]. In a similar vein, CNTs conjugated to paclitaxel demonstrated improved tumor apoptosis compared to free drug due to the CNTs ability to improve cell penetration of the drug, increasing intracellular concentration of paclitaxel by over 10 times. Moreover, the shelf life of paclitaxel was extended within the conjugated CNTs system by prolonging blood circulation of the drug. Significantly, no noticeable toxic effects were observed in mice [70]. Cisplatin in CNTs has also shown promise, a study by Ajima et al. reported that cisplatin conjugated with CNTs had 4–6 times increased anti-cancer efficiency compared to that of free cisplatin in mice [71].

### 2.4. Dual Drug Delivery and Synergistic Effects

Most promising of all is the prospect of the attachment of several drugs to a single vehicle wherein cooperation can be achieved. Given the highly adaptable nature of cancer, it is common practice for medical professionals treating cancer patients to prescribe several chemotherapy drugs to overcome drug resistances cancer strains may possess or develop [72]. It is for this reason that greater attention should be paid to the prospect of using C-dots as a vehicle towards delivering not just one but several drugs to the patient [73]. Poly(ethylene glycol)-conjugated MWCNTs has been shown to be an efficient drug carrier for overcoming multi-drug resistance [74]. Research has been done with micelles where poly(ethylene glycol) has been attached and used for dual drug delivery in several studies [73,75]. Similar research has advanced in other fields such as micelle delivery system wherein aspirin dispersed poly(vinyl alcohol) (PVA) and Dox were used in a drug delivery system [76]. Dual drug delivery systems are able to overcome poor therapeutic effect of single drug delivery system and achieve independent drug release, thereby maintain drug integrity and functionality. This effective delivery is made in large part possible by the pH differentiation found in endosomes containing lower pH than surrounding tissues [33]. The extra protons may allow for a higher number of drug agents to release from their carrier. In a study by Heister et al., CNTs were attached to both doxorubicin and mitoxantrone, which demonstrated enhanced ability to apoptose tumors as compared to either free drug alone; however, no ligand was used to improve the targeting efficiency [77]. Impressively graphene oxide nanocarriers have found success conjugating dox and camptothecin via π-π stacking and hydrophobic interactions. Remarkably improved toxicity in targeted carcinoma and breast cancer cells were found as compared to the single drug loaded version and thereby cell death, displaying possible synergistic effects [78]. Further work is merited specifically within Carbon nanoparticle systems given their unique capabilities as fluorescent platforms for ligand attachment, moreover C-dots are non-toxic when tested on urchin embryos unlike larger metal based nanoparticles such as ZnO that release toxic amounts of ROS [79]. Given the heterogeneity of quantum dots, most exibit some toxicity, although, the amount can vary heavily specifically within quantum dots such as CdSe and ZnS based quantum dots [80]. Different techniques can be used to reduce toxicity however, such as lipid coating.

Other areas of development may include using an anti-cancer drug alongside the new and lucrative field of small interfering RNA (siRNA) which shows much promise [81]. Given the relatively recent field of drug delivery, many reported dual and multi-stimuli responsive systems are proof-of-concept studies only, and thus are often not biodegradable, have low drug loading capacity, and may not work for in vivo applications [82].

## 3. Ligand-Receptor Mediated Delivery

Certain receptors can be overexpressed in different kinds of cancer cells; as a result, this overexpression can allow for the anticancer drug attached to ligands that can facilitate tumor penetration and do so in a more selective manner. Commonly overexpressed receptors in cancer cells include growth factor receptors (GFRs) [83]; however, these GFR ligands are difficult and costly to isolate for use in drug delivery systems. More commonly implemented ligands include transferrin, folic acid, and hyaluronan which are easily attainable and desirable for test projects due to their relatively inexpensive cost and ease of access. Although many of these receptors directly or indirectly induce cell growth and proliferation, there may be synergistic effects due to many chemotherapy drugs having short half-lives and causing DNA damage during replication; therefore, if receptor-induced proliferation occurs following ligand endocytosis, cellular apoptosis would be expected to occur due to the anticancer-agent’s action. This may also explain why single drug carrier systems often outperform the lone drug in causing tumor death. 

Carbon nanosystems, as previously discussed, possess the ability to be functionalized with different functional groups and even drugs or ligands [84]. The degree of functionalization can often be controlled by limiting the reaction time or amount of functional groups. And by controlling the degree of functionalization, the amount of drug delivered can be modulated [33]. Table 2, below, provides a list of the drug delivery vehicles discussed in this section. 

### 3.1. Transferrin-Based Targeted Delivery

**Figure 5:**
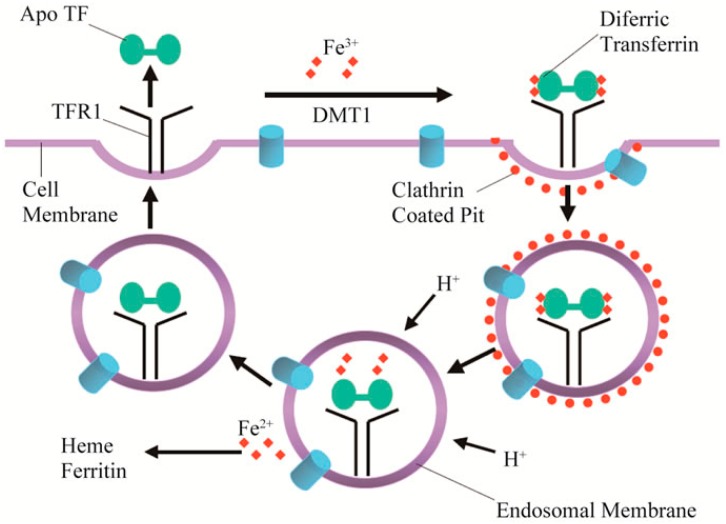
Diferric transferrin binds to transferrin receptor 1 at the cell surface, and the complex is endocytosed using clathrin-coated pits. Iron is released from transferrin and transported out of the endosome through divalent metal transporter 1 into the cytosol. Apotransferrin and transferrin receptor 1 return to the cell surface where they dissociate at neutral pH and are available for another iron cycle (reproduced with permission from [86]).

Iron is a critical component to many proteins, forming the heme groups, which are known for their ability to bind and transport oxygen. Iron is also needed for several metabolic processes, which include electron transport and deoxyribonucleic acid (DNA) synthesis [85]. Transferrin Receptor 1 is responsible for the uptake of iron into the cell via the use of clathrin-coated pits and, once iron is released, the receptors are recycled out alongside transferrin without iron bound (apotransferrin) as shown in Figure 5 [86]. Tumors in mice have been shown to have an increased uptake of transferrin [87]. Coupling DNA to transferrin via a carrier can serve as a potential alternative to common viral vector for gene therapy [88]. Transferrin is of particular interest when target tumors include areas of the brain. Transferrin can bypass the blood-brain barrier, a common obstacle for many other promising delivery vehicles [89]. Transferrin conjugated chemotherapy drugs such as Dox have been shown to be able to overcome multiple drug resistances in carcinoma, leukemia, and glioblastoma cell lines [90,91,92,93]. Transferrin shows growing promise in areas involving brain cancers as new drug delivery systems emerge. Within pediatric tumors, C-dots conjugated to Dox and transferrin were able to cause tumor death at higher instances compared to free drug alone and shown to bypass the blood-brain barrier [27,41]. Moreover, transferrin may prove especially lucrative for bioimaging due to its natural capacity to carry iron; as a result, MRI imaging may allow for a clearer tumor outline and thereby be of aid during surgical resection, especially when conjugated alongside magnetic C-dots [94].

Transferrin is a rather large protein with a mass of about 79 kDa. As a result, it can be easily purified via a size-exclusion column [26]. Transferrin can be readily conjugated via the commonly utilized EDC/NHS chemistry wherein the C-dots are functionalized and binding to an amine group within the transferrin polypeptide occurs. It should be noted that its exact binding location would be difficult to pinpoint, as there may be more than one; furthermore, depending on location of binding, transferrin may be entirely inactivated. Transferrin is not highly fluorescent with a small peak around 346 nm, which is much lower than most C-dots with broad peaks around 400 nm by comparison. Given its large size, characterization can be readily accomplished using mass spectroscopy. Matrix assisted laser desorption/ionization-time of flight (MALDI-TOF) can be used to prove a successful conjugation as well [26,41].

CNTs in lung cancer were able to use transferrin to achieve a 136 fold more efficient system as compared with free docetaxel alone, a chemotherapy agent [43]. However, CNTs with transferrin on its surface have been found to contribute to oxidative damage, which leads to cell death or possible tumor formation/progression [95]. Moreover, MWCNTs of 50 nm in diameter are known carcinogens and there are limited studies on the effects of drug delivery systems within normal cells [95]. Although several carbon-based nanoparticles are reported as nontoxic, this does not necessarily indicate they cannot contribute to tumor progression. Given that drug delivery vehicles improve drug efficacy and lower overall drug damage, a comparison between possible tumors caused by free CNTs, free chemotherapy agents, and CNTs based delivery systems would be interesting.

### 3.2. Folic Acid-Based Targeted Delivery

**Figure 6:**
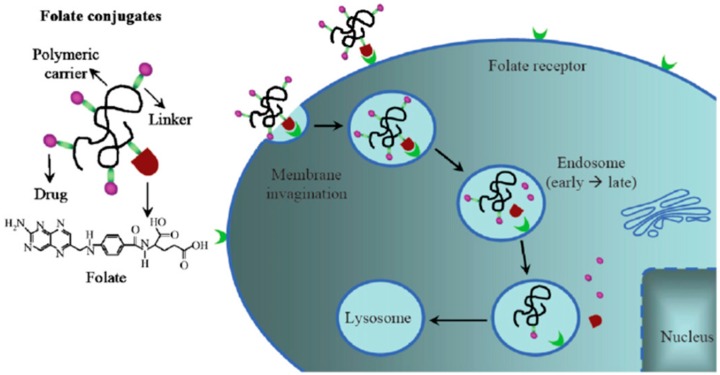
Folate receptor mediates binding of folic acid and endocytosis. The folate based conjugate, uses a polymeric carrier, such as C-dots, and a drug that is released following endocytosis (reproduced with permission from [96]).

Folic acid (FA) is readily sold and consumed as a B vitamin. FA plays a major role in the production of purines and pyrimidine synthesis and thereby can regulate cell division and growth [64]. Basic drug delivery mechanism shown using folate receptor targeting in Figure 6 wherein carbon nanoparticles could potentially serve the role of polymeric carriers to deliver anti-cancer drugs into the cell [96]. Leamon and Low are credited with conceiving the idea to use the folate receptor for targeted cell therapy and since a great quantity of research has emerged [97]. FA and conjugated FA bind with great specificity to the folate receptor shown in several studies using competition tests as described by Low et al. [98]. The folate receptor is overexpressed in epithelial, ovarian, cervical, breast, lung, kidney, colorectal, and brain tumors [99,100]. By comparison, sarcomas, lymphomas, and cancers of the pancreas, testicles, bladder, prostate, and liver usually do not show elevated levels of folate receptors. In normal tissues folate receptor expression is limited to the lungs, kidneys, placenta, and choroid plexus cells; wherein, receptors are found on the apical surface of polarized epithelia within the aforementioned cells [100].

C-dots functionalized with FA show great potential in detecting cancerous cells that overexpress FA as they are able to be endocytosed at a greater frequency as compared to normal cells [47,48]. In the same vein, C-dots when attached to an anti-cancer agent such as Dox show a similar ability with the added benefit of causing tumor death [46]. This specific system, interestingly, employed the use of bovine serum albumin to improve biocompatibility and increase drug loading [46]. Folate receptor is a more heavily varied receptor, unlike the more specialized transferrin receptor, with applications being found for drug delivery systems in a multitude of cancer studies. 

FA contains an amine attached to an aromatic ring as well as several carboxyl groups. Thus it is readily conjugated with the carboxylic rich C-dots once functionalized using EDC/NHS chemistry [34,47]. A similar approach has been implemented functionalizing FA with NHS and dicyclohexyl-carbodiimide (DCC) filtered, then mixed with C-dots at a pH of 10 [47]. A commonly used purification method is dialysis, as well as centrifugation. As for characterization, atomic force microscopy (AFM) and transmission electron microcopy (TEM) are common methods used to ensure a successful conjugation [34,47,53,54,55]. UV-Vis spectroscopy provides the most common method due to the very evident peak seen in FA at the 283 nm mark which would generally not overlap with most C-dots [34,47].

A 2011 study tested FA-functionalized MWCNTs magnetic nanoparticle hybrids as contrast agents and found that MWCNTs when conjugated to FA serve as ideal targeting agents for magnetic resonance imaging (MRI) [53]. In another study, MWCNTs were targeted to cancer cells *via* the folate receptor using a novel imaging approach. Confocal Raman microscopy was used unlike the typically used confocal fluorescence microscopy, which uses fluorescently labeled CNTs, the researchers were able to monitor cellular uptake in carcinoma cells [54]. Several studies back the efficacy of folate receptor when conjugated carbon nanoparticles and attached to a drug [34,47,53,54]. Interestingly, researcher found that using SWCNTs can cause cell destruction when conjugated with the folate receptor and exposed to near-infrared (NIR) light (700–1100 nm) due to excessive heating within carbon nanotube once aggregated near areas of high folate receptor density [55]. Overall, folate receptor is a very promising prospect for anti-cancer targeted drug delivery systems [101].

### 3.3. Hyaluronan-Based Targeted Delivery

**Figure 7:**
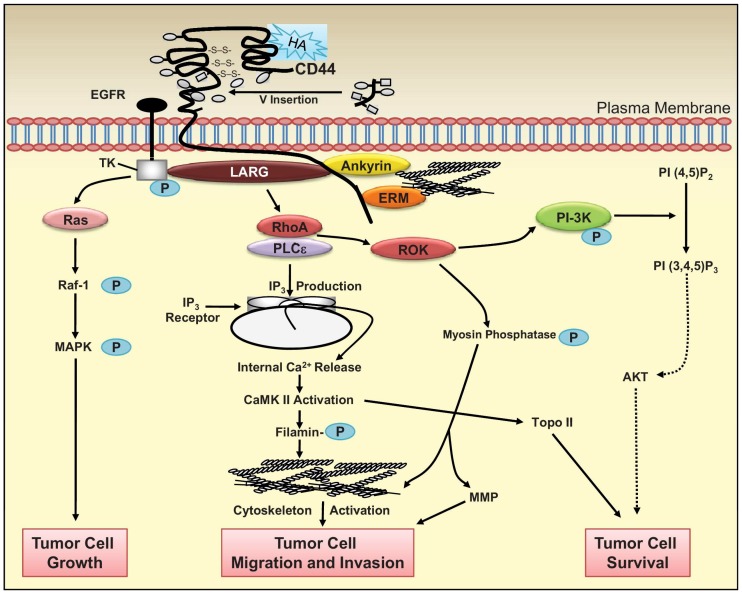
CD44 receptor binds hyaluronan (HA) causing a signaling cascade outlines above and eventual endocytosis (adapted with permission from [104]).

CD44 receptor binds hyaluronan or hyaluronic acid and functions as an adhesion regulator, where it is understood to function in hematopoiesis and lymphocyte activation [102]. Certain tumors that are otherwise mobile such as lymphomas have been shown to contain high levels of CD44 [102]. Not surprisingly hyaluronan is commonly sold for those with joint problems, as hyaluronan is a major component of the extracellular matrix and is a glycosaminoglycan that can be very large with a molecular weight at times reaching into the millions [103]. Studies have shown that for hyaluronan to interact with the CD44 receptor a minimum length of 6-8 units must be achieved and typically smaller sized conjugates work best. Hyaluronan has been shown to contribute significantly to cell replication and growth, migration, and can be frequently involved in the progression of some tumors as shown in Figure 7 due to its vast array pf downstream effectors [104,105]. Of particular interest is the downstream activation of Ras and phosphoinositide 3-kinase (PI-3K), two relatively well studied proteins found very frequently mutated in cancers [37]. This can prove a powerful ligand for targeting malignancies and as a marker [106,107]. CD44 has been found to be overexpressed in several cells such as breast cancers, intestinal cancers, colon cancers, leukemia prostate cancers, and pancreatic cancer due to its role in mediating receptor tyrosine kinases within the cell [108,109].

Recent studies have demonstrated that carbon nanoparticles can be used to effectively target cancer cells with CD44 overexpression when conjugated to hyaluronan [51,56,110,111]. Within C-dots, hyaluronan conjugated C-dots have shown the capacity to not only carry drugs but genes as well, thereby contributing to gene therapy as well as the usual cell imaging demonstrated by many studies [49]. The C-dots used in the study were synthesized from polyethylenimine; significantly, when toxicity between starting material and C-dots is compared, the C-dots show remarkably decreased toxicity, indicating C-dots as a potential way to decrease toxicity and maintain certain characteristics of desired material [49].

Hyaluronan contains several carboxylic groups that can serve for attachment of C-dots. In CNTs, common methods employ the use of DCC and ethylenediamine, whereby the surface for the nanotube is functionalized with amines. Then EDC and hyaluronan are added whereby conjugation is proven successful after brief dialysis [110]. C-dots by comparison first used hyaluronan activated by dopamine for attachment followed by dialysis [111]. Common methods to characterize a successful conjugation include NMR, FTIR, XPS, and to some extent fluorescence spectroscopy. Interestingly in FTIR several C–O–C bands of hyaluronan were observed after conjugation at 1046, 1078, 946, and 1150 cm^−1^, respectively [110]. Though this method of characterization may prove an issue since these readings fall in the fingerprinting region and different C-dots possess different fingerprinting regions, leading to possible misinterpretations.

Imaging in both in vivo using mice as well as in vitro has displayed a great possibility for CNTs within a photodynamic approach to treatment [110,111]. Such a method involves cell death by heating nanoparticles with infrared light and causing cells containing the carbon nanoparticle to apoptose via the activation of certain proteins such as heat shock proteins (hsp) and caspases. Interestingly researchers have found that hematoporphyrin monomethyl ether when attached to carbon nanoparticles and hyaluronan can be used for both photodynamic and photothermal therapy, thereby producing synergistic effects [110]. Furthermore, some of these studies have shown that when used in conjunction with an anti-cancer drug, tumor metastasis is severely hindered or impeded and growth inhibition is increased in some cases by up to five times when compared to inhibition in free drug alone [51,56]. Challenges in using hyaluronan arise in its variability in size. Furthermore, while the targeted CD44 may indeed be overexpressed and effectively bind hyaluronan, other cells in the body use hyaluronan in areas such as the joints; therefore, clinical studies would prove a difficult accomplishment.

## 4. Summary and Outlook

Cancer is becoming an increasingly prominent forefront of research and to this end carbon nanoparticles can serve as a powerful system for drug delivery. As these fields grow, carbon-based nanoparticles have been shown to be an effective means towards a drug delivery system in the effort to combat cancer. To this end, C-dots alongside similar carbon particles are nontoxic, unlike several other heavier, metal-based nanoparticles, and they possess functional groups similar in number and quantity to that of polymers like polyethylene glycol (PEG) commonly implemented in the field. 

Continuing research will prove fruitful towards the development of an efficient drug delivery system, and carbon nanoparticles are becoming increasingly useful in targeted therapy. Dox and Gem studies have shown promising results when used in conjunction with carbon nanoparticles. These two chemotherapy agents haven been approved for medical use in 1974 and 1995, respectively, and are likely to be replaced by new emerging drugs currently under clinical trials [112]. Research into novel drugs is growing as information grows. Possible anti-cancer agents such as siRNA are proving effective in cancer treatment. New and possibly more lethal drugs will lead to greater need for C-dots in producing drug delivery systems, wherein drug dosages can be lowered due to discriminatory targeting of cancer; furthermore, successful targeting would lead to increased drug efficacy. Dual drug delivery systems, by contrast, are in their infancy and not fully developed. Few papers have been published as it relates to carbon nanoparticles and low drug loading onto carbon functional groups may prove a challenge. Nonetheless, the continuing development of dual drug delivery in C-dots will prove of great importance in the continuing fight against cancer.

## Figures and Tables

**Table 1:** Selective examples of carbon nanomaterials based drug-targeting systems discussed in this paper.

Carbon Nanoparticles	Drug Loaded	Ligand Attached	Cell Targeted	Characterization Method	Drug Loading	Reference
C-dots	Dox	Nuclear localization signal peptide	A549	AFM, TEM, XPS, UV-Vis, Fluorescence, Confocal, Flow cytometry, FTIR, NMR	-	[44]
C-dots	Dox	-	HeLa	UV-Vis, Fluorescence, XPS, TEM, FTIR, Zeta	-	[14]
C-dots	Dox	-	HeLa	UV-Vis; Zeta; DLS; PL; TEM	260%	[45]
C-dots	Dox	Transferrin	CHLA-266, SJGBM2	Fluorescence, UV-Vis, MALDI-TOF	-	[41]
C-dots	Dox	Folic acid	HeLa	FTIR, UV-Vis, Zeta	85.6%	[46]
C-dots	-	Folic acid	HeLa, NIH-3T3, MCF-7	Fluorescence, TEM, UV-Vis	-	[47]
C-dots	-	Folic acid	HepG-2	UV-Vis, Fluorescence, FTIR, TEM, XPS	-	[48]
C-dots	Gene	Hyaluronan	HeLa	FTIR, NMR, UV-Vis, Fluorescence, TEM	-	[49]
CNTs	Dox	-	SH-SY5Y, HT-29, HepG-2	FTIR, TEM		[33]
CNTs	Dox	Folic acid	HeLa, 3T3	UV-Vis, IR, TEM, Zeta	149.3%	[32]
CNTs	Dox	Folic acid	HeLa	UV-Vis, TEM	-	[34]
CNTs	Dox	Folic acid	-	UV-Vis, Fluorescence, FTIR, SEM	91%	[50]
CNTs	Dox	Hyaluronan	-	SEM, TEM, Zeta, FTIR	-	[51]
CNTs	Gem	Folic acid	Breast cancer cells	Electron microscopy, FT-IR, X-ray diffraction	-	[42]
CNTs	Gem	-		FT-IR, NMR	-	[52]
CNTs	Docetaxel	Transferrin	A549	AFM, FTIR, TEM, Zeta	-	[43]
CNTs	-	Folic acid	Hela	UV-Vis, TEM, Zeta	-	[53]
CNTs	-	Folic acid	T24	AFM, TEM, Raman spectra	-	[54]
CNTs	-	Folic acid	HeLa	UV-Vis, AFM, Confocal, Fluorescence, SEM	-	[55]
CNTs	-	Hyaluronan	Gastric cancer stem cells	UV-Vis, Confocal, Flow Cytometry	-	[56]

**Table 2:** Selective ligand-mediated drug delivery systems discussed in this section.

Carbon Source	Drug Loaded	Ligand Attached	Targeted Cell	Reference
C-dots	Dox	Transferrin	CHLA-266, SJGBM2	[41]
C-dots	Dox	Folic acid	HeLa	[46]
C-dots	-	Folic acid	HeLa, NIH-3T3, MCF-7	[47]
CNTs	Docetaxel	Transferrin	A549	[43]
CNTs	Gem	Folic acid	Breast cancer cells	[42]
CNTs	-	Folic acid	Hela	[53]
CNTs	-	Folic acid	T24	[54]
CNTs	Dox	Folic acid	HeLa	[34]
CNTs	-	Folic acid	HeLa	[55]
CNTs	-	Hyaluronan	Gastric cancer stem cells	[56]
CNTs	Dox	Hyaluronan	-	[51]
